# Academic Procrastination: The Relationship Between Causal Attribution Styles and Behavioral Postponement

**Published:** 2011

**Authors:** Rahim Badri Gargari, Hossein Sabouri, Fatemeh Norzad

**Affiliations:** 1 Department of Psychology, University of Tabriz,; 2 Department of English & French Language and Literature of University of Tabriz

**Keywords:** Attribution, Controllability, Locus of control, Procrastination, Stability

## Abstract

**Objective:** This research was conducted to study the relationship between attribution and academic procrastination in University Students.

**Methods:** The subjects were 203 undergraduate students, 55 males and 148 females, selected from English and French language and literature students of Tabriz University. Data were gathered through Procrastination Assessment Scale-student (PASS) and Causal Dimension Scale (CDA) and were analyzed by multiple regression analysis (stepwise).

**Results:** The results showed that there was a meaningful and negative relation between the locus of control and controllability in success context and academic procrastination. Besides, a meaningful and positive relation was observed between the locus of control and stability in failure context and procrastination. It was also found that 17% of the variance of procrastination was accounted by linear combination of attributions.

**Conclusion:** We believe that causal attribution is a key in understanding procrastination in academic settings and is used by those who have the knowledge of Causal Attribution styles to organize their learning.

## Introduction

Procrastination may be defined as a way to avoid or escape from undesirable tasks. The procrastinator usually justifies his action by saying that "This is a boring assignment," or "I will do it later". In another definition, the procrastination has been described as an intentional, unreasonable postponement of the taken decisions, being unaware of the fact that this will bring about certain negative consequences (1). Procrastination is a widespread problem related to self-regulation, which is realized as deferral of the beginning and completing the important, necessary assignments. Procrastination may produce important negative consequences such as anxiety and depression, regarding the personality (2).

Depending on its cognitive, affective or behavioral components, procrastination will have different manifestations including academic, decisional, neurotic or compulsive procrastination. The most common form, however, is academic procrastination (3). It is defined as pervasive and permanent desire on the part of learner to postpone academic activities, which almost always is accompanied by anxiety. Deferring study to the night just before the exam and the accompanied anxiety and haste can be described as the most obvious and familiar instance of this form of procrastination (4). The studies tend to demonstrate procrastination as a behavioral problem among school and university students. Ferrari demonstrated that almost 20% of adults experience chronic procrastination, while the estimated rate of problematic academic procrastination among undergraduate students is at least 70-95 % (5). Another study estimated the serious and chronic procrastination among university students as 20-30% (6). 

Self-regulation is believed to be the conventional motivation explanation model in procrastination. Procrastinators actually fail in accomplishing self-regulation. Compared with normal students, they have lower ability to resist the social temptations, especially when the academic prize is rather far-off. Dweitte and Schouwenburg relate the dynamic character of the procrastination to temporal discount. Procrastination, to them, is a desire to undervalue distant prizes (7). For instance, we might postpone preparation for an exam since the prizes such as promoting to higher rank or avoiding a lower place in class lies far away from us. Setting objectives and designing a practical plan for intentions as an element of self-regulation was studied by Howell, Watson, Powell and Buro (8). Their results suggested that devising a practical plan for intentions such as time, place and the procedure correlates negatively with procrastination. 

According to the Theory of Planned Behavior, intention and decision are among the close characteristics to behavior performance, and mediate the effect of attitudes and thoughts on behavior (9). The recent studies confirmed that no differences exist between procrastinators and those not suffering from this problem in terms of deciding to act. For example, according to the experimental data, there is no diversity between procrastinators and normal individuals in terms of taking decision to find a job, to do academic assignments, and how many times they decide to study (9). It is apparent that actualization of these decisions and intentions is influenced by motivation factors.

"Causal Attribution" is suggested as being one of the motivation theories manipulating the realization of decisions. It refers to a process, in which individuals try to decide on the causal factors of an event or consequence. Individual’s response to a certain event is dependent on their interpretation of that event. Facilitating events prediction and control, the attributions explain the sensations, attitudes and behaviors and not only affect emotions but also manipulate individual's function (10). According to Weiner, behavioral explanations can influence both internal (readiness, emotion, decision) and external (others' behavior or environment) factors, which may be controllable or uncontrollable, and stable or unstable (11).

In internal causal explanation, the individuals correlate the events and consequences of the behavior with the ability, while in external causal explanation they relate it with external situations, luck or coincidence. As a rule, individuals attribute their success to internal willingness to shoulder the responsibility; but in case of failure, they blame external factors such as the difficulty level of the task. There exists, then, a sort of self-service in individuals. This supportive mechanism is there to provide the individual with a positive self-concept regardless of failure or success. To facilitate self-service attributions and support positive self-concept, individuals may recourse to rationalization. The process is called self-handicapping, through which the poor performance is justified. Self-handicapping is among the several self-defeating mechanisms that individuals employ to support positive self-concept when they fail to complete the assignment. Procrastinators avail themselves of this self-handicapping process. These students select the assignments that include more hinders that prevent their accomplishment or create situations that offer no way to success. For example, the self-handicapping students go for the assignments that are beyond their capacity or study in a crowded, bustling place. In this case, they create hinders for themselves that thwart their success. Actually, they provide thus external attributions for their failure, such as the place of study or a noisy class. Otherwise, these procrastinators postpone their assignments in order to support their self-respect through shunning failure. In addition, through the strategy of presenting themselves to other individuals-i.e. equipped with external reasons for their dilatory behaviors-, they guarantee that others will evaluate them as positive persons (12).

Procrastination can also be a function of the control attributions. Control attribution refers to the fact that to what extent individuals hold back the important consequences of their existence. The idea that one can direct his academic success (i.e. studying will result in higher mark) will facilitate his actual control over the events related to success (e.g. starting to prepare for exam). There is no consistent data concerning the role of attributions in procrastination. The results of Dweiite and Schouwenburg (7), Ferrari, Parker and Ware (13), and Steel, Brothen and Wambach (14) showed that there was no relation between control source and procrastination. Rothblum, Solomon and Murakami (15), however, demonstrated that procrastinators attribute passing the exams to external factors more that the other students. Trice and Milton (16) concluded that the procrastinators have a more external control source than non-procrastinators. The results of Janssen and Carton (17) suggested that the examinees with external control source are more reluctant to complete the assignments on time compared with those with internal source control. Carden, Bryant and Moss (18) reported that procrastinating students had more external control by comparison with normal ones. Finally, Howell et al. (8) revealed a significant negative correlation between procrastination and supposed academic control of students.

Ahadi (19) further revealed that students' attributions relate to their expectation of the future performance. His findings suggest that when students deem themselves a capable individual and attribute their failures to controllable factors (such as insufficient effort or knowledge), they probably concentrate on strategies that guarantee their future success. However, their motivation problems emerge when they attribute their failures to stable and uncontrollable factors since in this case they will be faced with decreased motivation to attempt and increased expectance of failure in future.

Given the very high prevalence of procrastination among university students (5, 6) and the role of causal attributions in sentimental and emotional consequences (11) and regarding the inconsistent results of studies on the relations between causal explanations and procrastination (7,8,12,13,15-18), the present study has this question as its main problem focus: Is there any relationship between causal explanations of success and failure, and academic procrastination of the students studying in department of foreign languages in University of Tabriz?

## Materials and Methods

*Subjects*

The statistical universe of the study included the students attending Department of English & French Language and Literature of University of Tabriz. According to Tablet of Morgan and Krijcie (20), 203 students were selected as study sample. Given the statistical population distribution model, the stratified proportional sampling method was selected. Based on proportion of the students in each department, 114 students from English and 89 from French Department were sampled (148 women and 55 men with an age range of 18 to 29). To consider ethical issues and increase the response rate, the students were asked to take part in the study anonymously.

*Instruments*

*a) Procrastination Assessment Scale-Student* *(SPSS):* Originally designed by Solomon and Rothblum (3) to analyze the academic procrastination in three areas of assignment preparation, preparation for exam and preparation of semester report, the scale consists of 21 items. Each item offered four options ranging from Rare (1 point) to Almost always (4 points). Jowkar and Aghapour (3) have translated it into Farsi. The factorial analysis by them revealed a general factor in procrastination questionnaire and its reliability coefficient estimated by Cronbach's alpha method is reported as 0.91 (3).

*b) Weiner's Causal Dimension Scale (CDA):* The earliest version of the scale was written by Russell in 1982. Ifthikhar Su'adi has translated it into Farsi as well as he has done well on psychometric characteristics (21). The questionnaire consists of sixteen supposed situations, eight of which are based success and the other eight on failure. The examinees are asked in this questionnaire to imagine themselves in each of these situations and think about the provided reason. They, then, go to answer the questions on that reason. The same nine questions are asked in all of the sixteen situations. Questions 1, 5 and 7 measure the control source and question 3, 6 and 8 the stability; questions 2, 4 and 9 assess with controllability of the causal attributions. In 1376, Iftikhar Su'adi examined the reliability of the scale in terms of semantic differential of each material using variance analysis method. The estimated main effect of control source proved to be 50-56% variance, that of the stability 14-19% and that of controllability 14-26%. Therefore, each of the three aspects is sufficiently measured with the nine-point discrimination scale and the resulted coefficients are in the significant level of 0.05. To measure the reliability of Weiner's evaluation of the causal dimensions Cronbach's alpha coefficient was used. The result was 0.75 for control source, 0.86 for stability and controllability 0.86 (21).

*Method of implementation and data collection*

By prior arrangement with the department's staff, one of the researchers attended the classes to help students in filling the questionnaires if required. The SPSS version 16 was used for the analysis of data. According to the nature of the variables, the present study used the step by step regression analysis. To test research hypothesis based on the role of causal attribution styles on behavioral postponement, at first assumption of tests centered on being normal, distribute linear, and homogeneous variance have been investigated with Kolmogrov-Smirnov test, histogram, and M box test respectively.

## Results

The correlation matrix of the study and the descriptive results are given in table 1. As it is clear from the table, certain independent variables of the study correlate significantly with procrastination. Thus, one sees is a negative significant relationship between procrastination and causal description of control source, and a positive one between procrastination and causal explanation of the failure reasons. In other words, to the extent that the students relate their success to internal factors such as their personal ability, to the same extent their procrastination decreases and similarly to the extent they relate failures to stable factors, their procrastination increases.

In order to explain the academic procrastination through factors related to causal explanation, the present study used the step by step regression analysis. The predictor variables introduced into the equation in order of the degree of their relationship with criterion variables, and those which did not significantly contribute to increased precision of predicting the criterion variable were deleted. The results of the last phase are given in tables 2 and 3.

The data in tables 3 and 4 are indicative of higher prediction ability for explanations of success control source, persistence in failure and controllability in success among the factors affecting academic procrastination in university students. Besides, in general, one may say that these factors explain 17% of common variance of procrastination. Given the regression coefficient and the significance level, it is clear that two sorts of causal explanations-i.e. success control source and controllability in success- have negative relationship and the other two explanations- namely persistence in failure and control source in failure- have a positive and significant relationship with procrastination. Put it simply, students attributing their success to internal and controllable factors showed a lower degree of procrastination in their academic activities while those relating their failures to internal, stable factors experienced high degree of procrastination.

**Table 1:** Descriptive statistics and interco relation matrix for key variables in the study

		**M**	**SD**	**1**	**2**	**3**	**4**	**5**	**6**
1	procrastination	38.03	13.10						
2	Locus of control in success	138.26	18.09	-.329*					
3	Locus of control in failure	113.51	16.57	.258*	.233*				
4	Controllability in success	104.26	19.39	-.272*	.080	-.145			
5	Controllability in failure	87.60	22.24	.239*	-.261*	-.197*	.355*		
6	Stability in success	147.20	22.21	.002	.227*	.075	-.308*	-.343*	
7	Stability in failure	134.30	19.23	.267*	.223*	.202*	-.194	-.187	.624*

*P≤0.05

**Table 2:** Hierarchical regression analyses of attributions predicting procrastination

variable	source	SS	D.F	MS	F	P
attributions	regression residual total	5970 28896.8 34866.9	4 199 203	1492.5 145.2	10.2	.001*

*P≤0.05

**Table 3:** Standardized b coefficient and t values for the predictors of the hierarchical regressions

variable	b	ß	t	p	R2
Locus of control in success	-.244	-.366	-4.881	.001	%17
Stability in failure	.136	.230	3.250	.001	
Locus of control in failure	.116	.147	2.194	.029	
Controllability in success	-.089	-.146	-2.107	.036

## Discussion

The study aimed to determine the role of causal explanations of students in their success and failure in their academic procrastination. In this regard, step by step regression of causal explanation factors in relation with academic procrastination was measured. The method was used because, first, the insignificant variables are deleted and, second, in each step the effect of the previous variables are reexamined in terms of significance.

The relationship between students' control source and procrastination was one of the findings of the current work. In other words, there was a negative relationship between procrastination and control source in successful instances and a positive correlation between procrastination and the control source in failures. Hence, students that attributed their success to internal factors -i.e. their attempt and abilities- showed a lower degree of procrastination and those associating their negative academic results with internal (disability) factors demonstrated a higher degree of procrastination in completing their academic assignments. Carden et al (18) reported that procrastinating students had a higher external control compared with normal students. Janssen and Carton (17) showed that examinees with internal control source were more eager to finish the assignments punctually than those with external control source. The results of Beck, Koons, and Milgrim (22) suggested that students with internal control source showed a lower degree of procrastination, which contradicted data from some studies (7,13).

Results from the present study were consistent with Rotter's social learning theory prediction of individuals' behavioral differences with expectations of internal and external control source (23). Students who attributed the result and consequences to external factors such as luck and fate more often postponed academic assignments than those who felt correlation between their behavior and the final outcome.

According to Weiner (24), source is generally related to pride and self-respect or self-value. If students relate the academic accomplishment to certain internal factors, it will result in their increased sense of value and this in turn will contribute to more desirable behavioral and functional outcome such as decreased procrastination. However, students who have external causal explanations such as task's level of difficulty in case of failure try to provide some self-serving for themselves. The reason for this supportive self-service is that it provides them with a positive self-concept in spite of the failure. Procrastinating and postponing the academic assignments by these students is a way to escape the failure and support the self-respect, and according to Ferrari (12) is a way of self-handicapping.

Of the other findings of the study was that the students relating their success to controllable factors such as hardworking completed their assignments on time and never postponed its beginning, continuation or completion. Some of the findings by other scholars are consistent with this observation. Howell et al. (8) showed a significant negative correlation between procrastination and student's imaginary academic control. Ahadi (19) demonstrated that when students attribute their failure to controllable factors (such as insufficient endeavor or knowledge), they may concentrate on strategies that guarantee their future success. Their conative problems emerge, however, when they correlate their failures with stable and uncontrollable factors. In this case, their effort motivation is decreased and the expectation for future failure is increased.

According to Weiner's attribution theory (24), reason controllability is a function of personal responsibility. If a reason is controllable, the individual suppose himself as a responsible person, while uncontrollable reasons lead to decreased sense of responsibility for consequences. According to this theory, when students relate their success to controllable factors such as hardworking, they regard the changes in environment as a function of their own actions. This causal explanation provides them with energy and power. Hence, they define realistic objectives for themselves and realize them and frequently evaluate their progress.

Finally, the current study revealed that stable causal explanation in failures correlates positively and significantly with procrastination. In other words, students who blamed stable factors (such as aptitude and capability) for their failures displayed more procrastination. In their analysis on children's depression, Abela and Zachary (25) reported that the children tended to correlate negative consequences with stable factors. Nouri Qasemabadi (26) showed that following the light academic failure stress unsuccessful individuals resort to internal, general and stable causal attribution.

In explaining the present finding on the basis of Weiner's view (24), one can say that the stability aspect correlates with hope and disappointment (subsequent expectations). Relating a consequence to a stable factor may create the expectation that the same outcome will occur while relating it with an unstable factor will raise the expectation that the following consequences will be different from those preceded. So, it is possible to say that students who reprimanded stable factors for their failure underwent some sort of disappointment. They had an uncertainty about successfully doing their assignments and thus, failed to start, resume and complete them punctually. Several studies (27) have emphasized the correlation between expectancy and achievement tests marks, course ranks in high school and academic levels. Specifically, lower expectancy level has been shown to be a predicator of insufficient exam anxiety levels, study methods and exam strategies.

Given the findings of the present study on the role of students' causal explanations in their procrastination, it is suggested that the university staff should facilitate the creation of conformable attribution on the part of students through giving feedback of their performance. The other suggestion offered by the present study to the practitioners of counseling centers of universities is that they can hold attribution re-education courses through educational workshops for students involved with procrastination behavioral problem. As a part of their objectives, these workshops will set to help students challenge the unconformable attribution patterns and acquire conformable patterns.

Of the limitations of the current study one can mention the use of self-reporting tool in procrastination. Using such a tool, the students will be influenced by factors including desirability and social acceptance. Therefore, in order to prevent biased measuring tool it is suggested that the future behavioral studies on assignments and procrastination employ experimental measuring tool.

## Authors' Contributions

RBG conceived and designed the evaluation and helped to draft the manuscript and performed parts of the statistical analysis. HS participated in designing the evaluation and helped to draft the manuscript. FN collected the clinical data, interpreted them and revised the manuscript. All authors read and approved the final manuscript.

## Notes

**Declaration of Interest:** None.

## References

[1] Simpson W, Pychyl T (2009). In search of the arousal procrastinator: Investigating the relation between procrastination, arousal-based personality traits and beliefs about procrastination motivations. Personality and Individual Differences.

[2] Sirois F (2007). ‘‘I’ll look after my health, later’’: A replication and extension of the procrastination–health model with community-dwelling adults. Personality and Individual Differences.

[3] Jowkar B, Delavarpur MA (2006). J of new education ideas.

[4] Solomon LJ, Rothblum ED (1984). Academic procrastination: Frequency and cognitive-behavioral correlates. J couns psychol.

[5] Ferrari JR (2001). Procrastination as self-regulation failure of performance: Effects of cognitive load, self awareness, and time limits on ‘working best under pressure’. Eur J Pers.

[6] Steel P (2007). The nature of procrastination: A meta-analytic and theoretical review of quintessential self-regulatory failure. Psychol Bull.

[7] Dewitte S, Schouwenburg HC (2002). Procrastination, temptations, and incentives: The struggle between the present and the future in procrastinators and the punctual. Eur J Pers.

[8] Howell A, Watson D (2007). Procrastination: Associations with achievement goal orientation and learning strategies. Person Indiv Differ.

[9] Sirois F (2004). Procrastination and intentions to perform health behaviors: The role of self-efficacy and the consideration of future consequences. Pers Indiv Differ.

[10] Mahboudi M (1999). [The comparison of causal attribution styles of special women (prostitution) with normal women.] [MA. Dissertation].

[11] Weiner B (1985). Spontaneous causal thinking. Psychol Bull.

[12] Ferrari JR (1991). Compulsive procrastination: Some self-reported characteristics. Psychol Rep.

[13] Ferrari JR, Parker T, Ware C (1992). Academic procrastination: Personality correlates with Myers-Brggs types, self-efficacy, and locus of control. J Soc Behav Pers.

[14] Steel P, Brothen T, Wambach C (2001). Procrastination and personality, performance, and mood. Person Indiv Differ.

[15] Rothbloom E, Solomon L, Murakami J (1986). Affective, cognitive, and behavioral differences between high and low procrastinators. J couns psychol.

[16] Trice A, Milton C (1987). Locus of control as a predictor of procrastination among adults in correspondence course. Percept Mot Skills.

[17] Janssen T, Carton J (1999). The locus of control and task difficulty on procrastination. J Genet Psychol.

[18] Carden R, Bryant C, Moss R (2004). Locus of Control, Test anxiety, academic procrastination, and achievement among college students. Psychol Rep.

[19] Ahadi H (1994). J of Edu.

[20] Krejcie RV, Morgan DW (1970). Determining sample size for research activities. Educational and Psychological Measurement.

[21] Ifthikhar Su'adi Z (1999). [The survey of relationship between attribution styles, self-esteem, and social-economical status with achievement performance.] [Dissertation].

[22] Beck BL, Koons SR, Milgrim DL (2000). Correlates and consequences of behavioral procrastination: The Effects of Academic Procrastination Self-consciousness. Self-esteem, and self-handicapping. J Soc Behav Pers.

[23] Pintrich PR, Schunk DH (2002). Motivation in education: Theory, research, and applications.

[24] Weiner B (1994). Integrating social and personal theories of achievement striving. Rev Educ Res.

[25] Abela J, Zachary R (1999). The helplessness of depression. [Dissertation] Abstracts International: Section B: The science and engineering.

[26] Nouri GR (1993). [Investigation of cognitive-stress model from depression.] [.Dissertation.].

[27] Alexander ES, Onwuegbuzie AJ (2007). Pers Indiv Differ.

